# Epidemiological and nursing perspectives on insulin-derived amyloidosis: a scoping review

**DOI:** 10.20407/fmj.2026-009

**Published:** 2026-09-25

**Authors:** Fumiya Hisano, Kimie Takehara, Keisuke Nakanishi, Miharu Tamaoki, Toshihiko Yanagita, Junko Sugama

**Affiliations:** 1 Department of Nursing Science, Graduate School of Medicine, Nagoya University, Nagoya, Aichi, Japan; 2 Faculty of Nursing, School of Health Sciences, Fujita Health University, Toyoake, Aichi, Japan; 3 Department of Clinical Pharmacology, School of Nursing, Faculty of Medicine, University of Miyazaki, Miyazaki, Miyazaki, Japan

**Keywords:** Diabetes, Epidemiology, Injection-site complications, Insulin-derived amyloidosis, Nursing

## Abstract

**Objectives:**

Insulin-related amyloidosis (IDA) is a subcutaneous complication that may interfere with insulin absorption. Although early detection may prevent the deterioration of glycemic control, this condition is often overlooked. In this scoping review, we aimed to summarize the epidemiological characteristics of IDA and identify future research challenges from a nursing perspective.

**Methods:**

Following the Preferred Reporting Items for Systematic Reviews and Meta-Analyses extension for scoping reviews guidelines, a comprehensive literature search was conducted using PubMed, Scopus, Web of Science, and the Japan Medical Abstracts Society database. The eligible studies comprised original articles and case reports regarding patients with histopathologically confirmed IDA published in English or Japanese between January 2009 and August 2022.

**Results:**

No epidemiological research articles, but a total of 29 case-based reports regarding 38 patients, were identified. Most of the patients were male (n=29), they were aged 27–87 years, and both type 1 and type 2 diabetes were represented. The abdominal region was the most common site of lesions (n=23). Histopathology was the primary diagnostic method, although some patients were also evaluated using diagnostic imaging. Many of the patients noticed subcutaneous masses but delayed seeking medical care, and some continued injecting insulin into the affected sites. Medical consultations were often prompted by poor glycemic control or complications of diabetes.

**Conclusions:**

This review highlights the paucity of epidemiological research on IDA and underscores the importance of early detection and education. Limited awareness regarding injection-site abnormalities and the lack of early assessments may contribute to delays in diagnosis.

## Introduction

Insulin-derived amyloidosis (IDA) is a dermatological complication of insulin therapy that develops when amyloid accumulates subcutaneously, owing to repeated insulin injection at a single site over the long term.^1,2^ IDA typically presents as firm subcutaneous nodules at the injection site. Insulin injection into IDA lesions results in impaired insulin absorption and therefore poorer glycemic control.^3^

To prevent IDA, patients should rotate the injection sites used and avoid injecting at the same location. However, where this advice is not heeded, insulin nodules can develop, and these represent an often overlooked potential cause of poor glycemic control. This may suggest inadequate levels of early IDA detection and patient education, despite the fact that early detection may help prevent the deterioration of glycemic control.

Nurses who instruct patients regarding insulin injection techniques and provide ongoing management play a critical role in the early detection and management of IDA. To establish a system that enables nurses to identify IDA early, it is necessary to collate and organize the existing knowledge regarding this condition, including epidemiological information, its clinical features, the characteristics of the patients, and the reasons for consulting a medical professional, with a nursing perspective in mind.

Although research reviews of IDA have previously been performed, they have important limitations. First, most of these studies focused primarily on the perspective of physicians, and no epidemiological review has been conducted from a nursing standpoint. Second, IDA is sometimes confused with insulin-induced lipohypertrophy, and in some previous studies the two conditions have not been clearly distinguished. Lipohypertrophy is a distinct condition that is characterized by soft, painless subcutaneous nodules at injection sites that generally resolve spontaneously with proper site rotation.^4^ Therefore, the differentiation of IDA from lipohypertrophy is essential to ensure early detection and provide appropriate care. Furthermore, despite the numerous Japanese studies published on “insulin balls,” a colloquial term for IDA introduced by Nagase et al., previous reviews have not incorporated studies published in Japanese.^5^

To address these issues, in the present study, we aimed to summarize the reported epidemiological characteristics of IDA by reviewing the literature in both English and Japanese and to identify future research challenges from a nursing perspective.

## Methods

### Study design and methodology

We conducted a scoping review in accordance with the Preferred Reporting Items for Systematic Reviews and Meta-Analyses extension for scoping reviews guidelines.^6^ Ethics approval was not required because the study involved a secondary analysis of published data only. The authors declare that they have no competing interests.

### Aims of the scoping review

In this review, we aimed to identify (1) published epidemiological studies on IDA, and to record the names of the authors, the publication year, the country of origin, and the journal field; (2) the demographic characteristics of patients with IDA, including their age, sex, type of diabetes, history of insulin use, and the diagnostic methods and treatments used; (3) the morphological features of IDA lesions, including their location, size, shape, and coloration; (4) the patients’ subjective symptoms, their level of awareness, and their reasons for seeking medical attention; and (5) gaps in the current education, assessment barriers, and nursing-related interventions in the literature.

### Eligibility criteria

A diagnosis of IDA was confirmed by histopathological examination of a skin biopsy. The eligible studies comprised original articles and case reports written in English or Japanese and published between January 1, 2009, and August 26, 2022. Review articles, meta-analyses, clinical guidelines, editorials, letters to the editor, conference notes/abstracts, posters, and oral presentations were excluded.

### Information sources

To identify relevant articles, searches were conducted of PubMed, Scopus, and Web of Science. Furthermore, the Japan Medical Abstracts Society database (Ichushi-web) was used to identify studies published in Japanese. The research team collaboratively developed the search strategy. The final search results were exported into Rayyan (https://www.rayyan.ai/), then duplicates were removed and the articles were further screened independently by two reviewers (NK and HF).

### Search strategy

Keywords and Medical Subject Headings (MeSH terms) related to “insulin balls” and IDA were used in combination with Boolean operators “AND” and “OR.” For original articles, the following search terms were used: ((insulin-derived amyloidosis) OR (“insulin ball”)) AND (epidemiology). For case reports, the following search terms were used: ((insulin-derived amyloidosis) OR (“insulin ball”)). The following filters were applied: (1) article type: case report; (2) Species: human; and (3) language: English or Japanese.

### Selection of sources

After the studies were imported into Rayyan, the two reviewers independently screened the titles and abstracts, and studies that clearly did not meet the inclusion criteria were excluded. Thereafter, three reviewers (NK, HF, and TK) independently assessed the full text of articles that were deemed to be potentially eligible on the basis of the predefined eligibility criteria. Disagreements during study selection were resolved through discussion.

### Data charting process

One author (NK) developed a data extraction form that was designed to capture key characteristics of each study and the features of the patients. The data were extracted by one author (HF) and verified by another author (TM), with any discrepancies in the extracted data being resolved through discussion.

### Data items

The following information was extracted from each study: (1) author name, year of publication, type of publication, country of origin, language, and journal field; (2) patient characteristics (age, sex, type of diabetes, history of insulin use, and the nature of the diagnostic examination and treatment); (3) morphological features of the lesion (location, size, shape, and color); and (4) subjective symptoms, patient awareness, and the reasons for consulting a medical professional.

## Results

### Study selection

A flowchart showing the process of selection of original articles is shown in Figure 1. The database searches initially identified 12 original articles, none of which were duplicates. However, after title and abstract screening, 11 studies were excluded, and the full text of the remaining study was reviewed. After reviewing the full text, this study was also excluded, and therefore no original articles on the epidemiological characteristics of IDA were included.

A flowchart showing the selection process for case reports is shown in Figure 2. Of the 79 records identified from the databases, 64 remained after the removal of duplicates. After title screening, a further 31 reports were excluded, and the full text of the remaining 33 were assessed. Ultimately, 29 eligible reports were included in the analysis.^7–32^

### Characteristics of the included studies

Table 1 summarizes the details of the included reports, including the author names, year of publication, country of origin, and journal field. Twenty-nine reports (25 individual case reports and 4 case series) from six countries describing a total of 38 patients were selected. Of these, 18 were written in English and 11 were written in Japanese. The most frequent journal category was dermatology, followed by pathology, but general medicine, diabetology, endocrinology, and plastic surgery were also represented.

### Synthesis of the results

Table 2 shows the epidemiological characteristics of the patients. Their ages ranged from 27 to 87 years, 29 were male and 9 were female, and 18 and 20 had types 1 and 2 diabetes, respectively. The duration of insulin use ranged from 4 to 32 years. Twenty-three patients were diagnosed using histopathology alone, and computed tomography (CT) and magnetic resonance imaging (MRI) were also used in four and three reports, respectively. In addition, blood tests and ultrasonography were used. Eleven patients underwent surgical excision of the lesion, and nine were managed by altering the insulin injection site. Other management approaches included patient education regarding proper insulin storage and topical ointment application.

### Morphological characteristics of the lesions

Table 3 summarizes the morphological characteristics of the IDA lesions. The most common anatomical site was the subcutaneous tissue overlying the abdomen, which was reported for 23 patients, but lesions in the upper and lower extremities were also reported. The lesions ranged from 10 to 130 mm in diameter in the 20 patients for whom such information was available. A pedunculated form was described by two patients. The colors of the lesions were reported for three patients; these were described as reddish-brown, brown to purplish-red, and grayish-white.

### Subjective symptoms, patient awareness, and reasons for consulting a medical professional

Table 4 summarizes reports the subjective symptoms, patient awareness, and reasons for seeking medical attention for the patients. The most common reasons for consulting a medical professional were poor glycemic control and complications of diabetes, such as hypoglycemic episodes, diabetic foot gangrene, and diabetic retinopathy (nine cases, 52.9%) (Figure 3). Six other patients sought medical attention after noticing a skin mass (35.3%), and one patient sought medical attention after suspecting IDA (5.9%).

All the patients were aware that a mass was present, although only one reported pain and bleeding associated with the lesion (6.7%). Four of the patients noticed the mass up to 5 years prior to their medical consultation (26.7%). The period of time between symptom recognition and clinical consultation varied. Notably, six patients continued injecting insulin into the same area despite being aware of the mass, citing the ease of pinching and minimal pain as reasons (85.7%).

The patients often consulted a medical professional because of a systemic problem, such as poor glycemic control or a complication of diabetes, rather than because of the IDA itself. The need for nursing interventions was categorized as either a lack of recognition of the clinical significance of the mass despite awareness of its presence, or behavioral factors such as continuing injections into the same site.

## Discussion

The present study is one of the first scoping reviews to investigate the epidemiological characteristics of IDA from a nursing perspective, based on data obtained during studies published in both English and Japanese. Despite the gradual increase in the number of publications regarding IDA in recent years, literature searches identified no original articles addressing the epidemiological aspects of IDA. Approximately half of the included case reports described Japanese patients, suggesting that there may be more active research on IDA in Japan than elsewhere, although there may also be ethnic or regional differences in incidence. However, it was not possible to confirm the explanation in the present study, and therefore this issue warrants further investigation. We found no obvious differences between Japanese patients and patients of other ethnicities in this limited dataset.

When we integrated the findings related to the patient characteristics, morphological features, and consultation behaviors, three key themes were identified: educational gaps, assessment barriers, and nursing-related implications.

Regarding the educational gaps, all the patients were aware of the presence of a subcutaneous mass; however, only one recognized this as being related to IDA.^20^ This suggests that although patients can identify physical changes at injection sites, they may lack an understanding of their clinical significance. As a result, IDA may not be perceived as a condition that requires medical evaluation. In addition, a large proportion of the patients continued injecting insulin into the same site, despite being aware of a mass (85.7%). Thus, there was a disconnect between their knowledge and the practices implemented.

Regarding assessment barriers, these may be categorized as follows. First, at the system and clinician level, the use of histopathological confirmation as the definitive diagnostic method may limit early detection and contribute to the under-recognition of IDA in routine clinical practice. Although the use of complementary diagnostic techniques (CT, MRI, ultrasonography, and blood testing) has been reported, no standardized noninvasive diagnostic strategy has been established. In addition, variability in lesion morphology may further hinder early detection. Although most lesions are located in the abdominal region and have relatively consistent characteristics, some have atypical features, such as pedunculated morphology or unusual coloration, which may complicate visual recognition during routine assessments.^10,14,24,27^ These factors may contribute to the lack of early identification. Collectively, these findings emphasize the importance of improving the awareness of healthcare professionals, and particularly of nurses, who often provide guidance regarding insulin injections and site monitoring.

Second, at the patient level, there may be insufficient recognition of the clinical importance of injection-site abnormalities, despite awareness of their existence. This disconnect between awareness and clinical interpretation may contribute to delays in seeking medical attention. In fact, medical professionals were most frequently consulted because of systemic complications of diabetes, such as poor glycemic control, hypoglycemic episodes, and diabetic foot gangrene, rather than because of the presence of IDA itself. This indicates that IDA is often not recognized as being important enough to warrant medical attention, even when a mass is detected. As a result, delays in seeking medical care were common, with some patients recognizing the lesion up to 5 years prior to their consultation. These findings suggest that IDA is frequently under-recognized by patients and during routine clinical assessments, resulting in delays to diagnosis.

Regarding nursing-related interventions, the persistence of inappropriate injection-site use and delays in seeking help may indicate that there are deficiencies in current educational approaches. Although injection-site rotation is a well-established component of education regarding diabetes self-management, the present findings highlight the need to support the translation of knowledge into the most appropriate self-management practices. Furthermore, the fact that the patients did not typically consult medical professionals regarding their IDA suggests that the existing educational approaches may not sufficiently emphasize the clinical consequences of injection-site abnormalities or the need for the timely escalation of care. Therefore, more structured and reinforced educational interventions may be beneficial in not only providing information, but also promoting appropriate behaviors. In addition, the findings highlight the importance of developing more effective strategies for the early detection of IDA. Enhancing the ability of nurses to recognize atypical morphological features, together with improvements to routine assessment practices, may contribute to earlier identification of lesions and interventions. Integrating patient education with improvements in clinical assessment approaches may also play an important role in reducing delays in diagnosis and management.

The present review has some limitations that warrant consideration. First, the included studies were limited to case reports and case series that described a small number of patients, and therefore there was the potential for selection bias. Thus, the findings may not reflect the true epidemiology of the condition in the diabetic population as a whole. Second, only studies of patients with histopathologically confirmed IDA were included, which may have led to the exclusion of relevant cases. Therefore, further large-scale studies are needed to clarify the epidemiological characteristics of IDA.

In conclusion, we have highlighted the lack of epidemiological research on IDA, showing that the existing evidence has largely been obtained through descriptive studies, and therefore a significant knowledge gap remains. Although patients may notice injection-site abnormalities, these are not always appropriately interpreted or promptly evaluated, contributing to delays in diagnosis. This may be explained by educational gaps and a lack of early assessments.

## Figures and Tables

**Figure 1:**
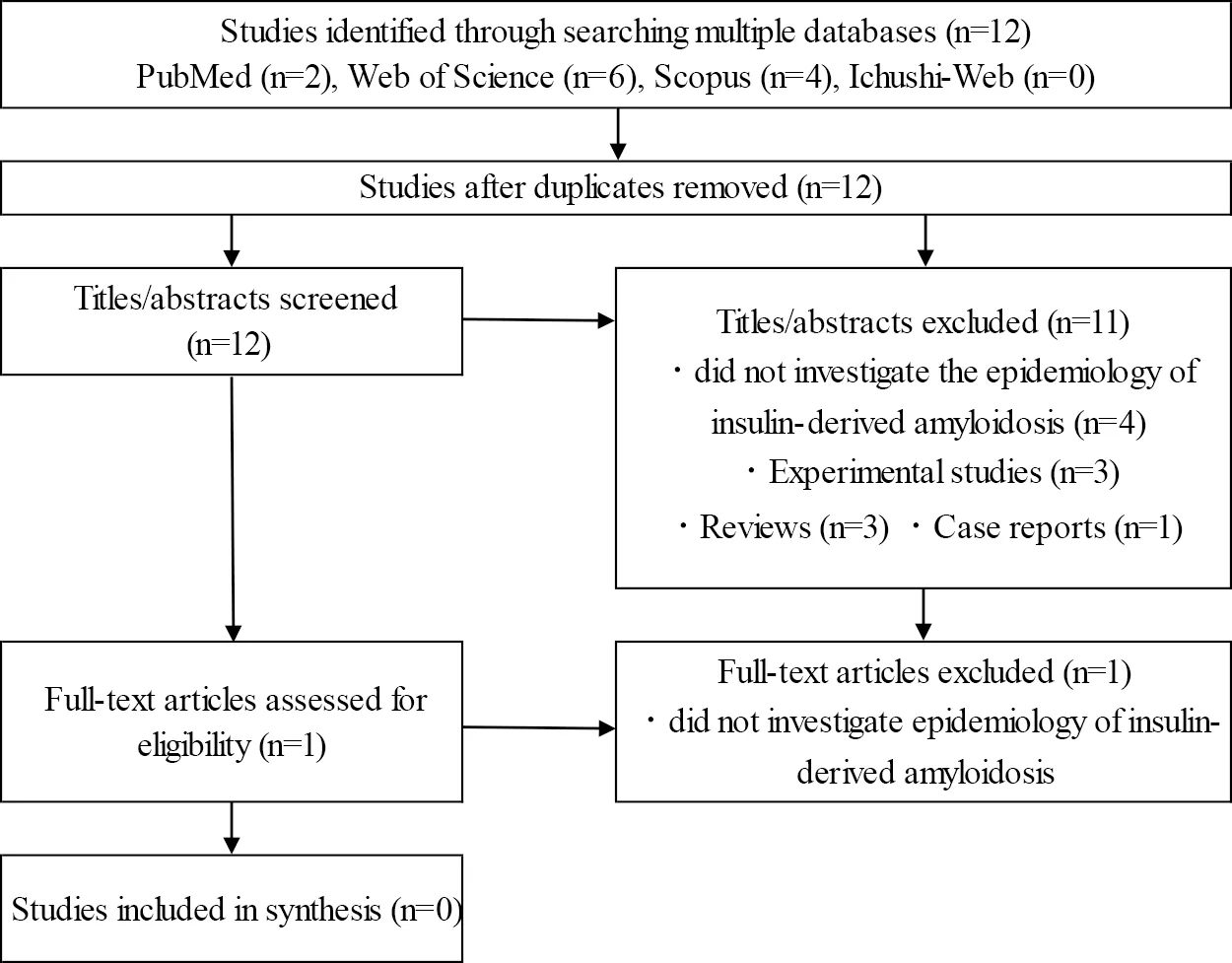
Selection process for original articles, following the Preferred Reporting Items for Systematic Reviews and Meta-Analyses extension for scoping reviews.

**Figure 2:**
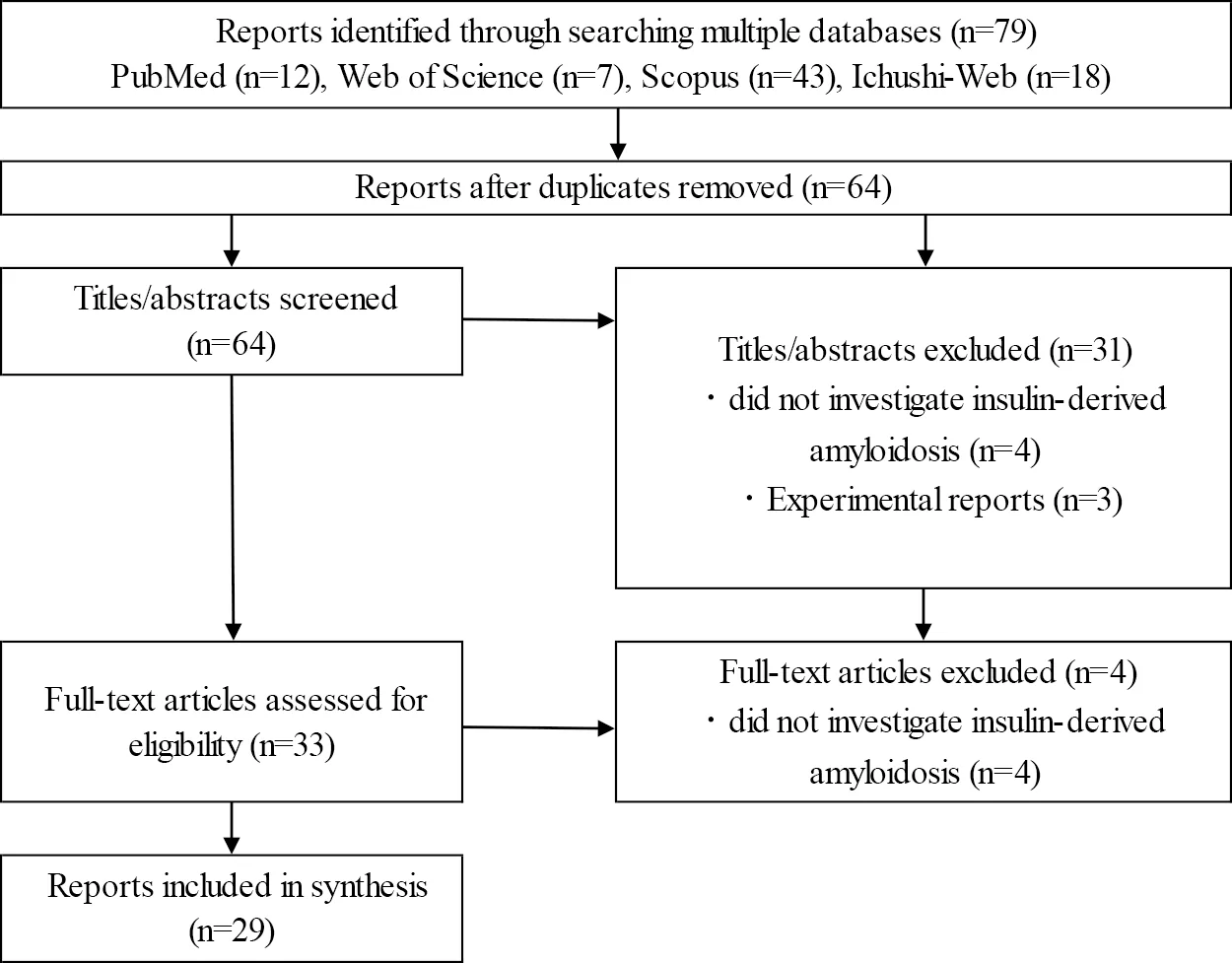
Selection process for case reports, following the Preferred Reporting Items for Systematic Reviews and Meta-Analyses extension for scoping reviews.

**Figure 3:**
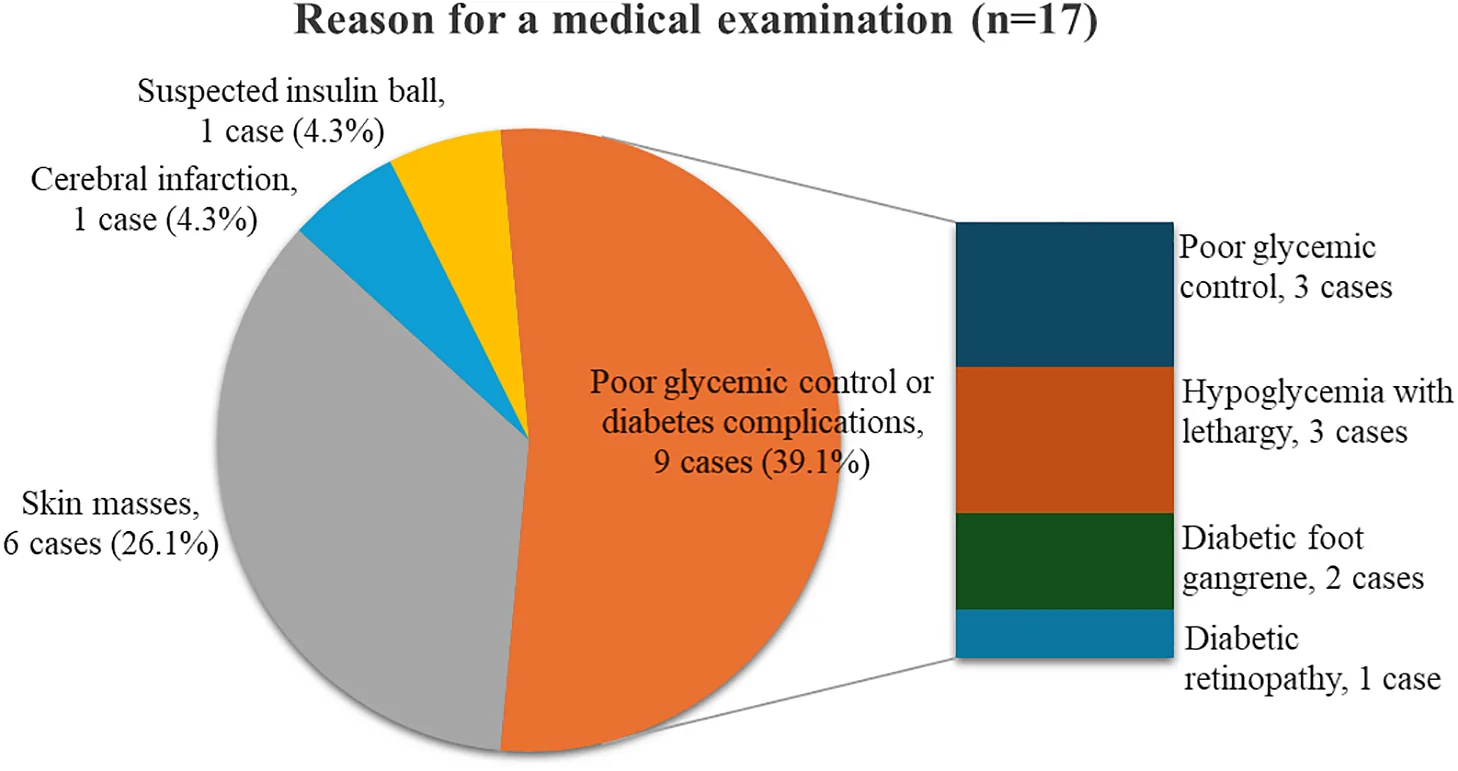
Reasons why the patients consulted a medical professional.

**Table 1:** Characteristics of the included sources

No.	Authors	Year	Type of publication	Country	Language	Journal field
1	Nagase T, et al.^5^	2009	Case report	Japan	English	Medicine
2	Yumlu S, et al.^7^	2009	Case series	United States	English	Pathology
3	Sie MPS, et al.^8^	2010	Case report	Netherlands	English	Medicine
4	Endo J, et al.^9^	2010	Case report	United States	English	Dermatology
5	Takao M, et al.^10^	2012	Case report	Japan	Japanese	Dermatology
6	Kudo-Watanuki S, et al.^11^	2013	Case report	England	English	Dermatology
7	Nagase T, et al.^3^	2014	Case series	Japan	English	Medicine
8	Nandeesh, BV, et al.^12^	2014	Case report	India	English	Pathology
9	Bernardez C, et al.^13^	2015	Case series	United States	English	Dermatology
10	Yabe S, et al.^14^	2015	Case report	Japan	Japanese	Diabetes
11	Grunes, D. et al.^15^	2015	Case report	United States	English	Pathology
12	Mangla, A, et al.^16^	2016	Case report	United States	English	Diabetes
13	Okamoto T, et al.^17^	2017	Case report	Japan	Japanese	Neurosurgery
14	Higuchi T, et al.^18^	2017	Case report	Japan	Japanese	Dermatology
15	Miyake M, et al.^19^	2018	Case report	Japan	Japanese	Dermatology
16	Utsunomiya R, et al.^20^	2018	Case report	Japan	Japanese	Dermatology
17	Tomita S, et al.^21^	2018	Case report	Japan	Japanese	Dermatology
18	Katzman BD, et al.^22^	2018	Case report	United States	English	Dermatology
19	Iwaya K, et al.^23^	2019	Case report	Japan	English	Endocrine
20	Terai S, et al.^24^	2019	Case report	Japan	Japanese	Dermatology
21	Tachibana M, et al.^25^	2019	Case report	Japan	Japanese	Pathology
22	Kitano Y, et al.^26^	2019	Case report	Japan	Japanese	Dermatology
23	Nagase T, et al.^1^	2020	Case series	Japan	English	Diabetes
24	Chen MC, et al.^27^	2020	Case report	England	English	Dermatology
25	Samlaska, et al.^28^	2020	Case report	United States	English	Dermatology
26	Carll T, et al.^29^	2020	Case report	United States	English	Dermatology
27	Miyata T, et al.^30^	2020	Case report	Japan	Japanese	Dermatology
28	Tummidi, S, et al.^31^	2021	Case report	India	English	Pathology
29	Vuillamy, C, et al.^32^	2022	Case report	France	English	Dermatology

**Table 2:** Epidemiological characteristics of the patients

Age (years), median (range)		61 (27–87)
Sex (n)	Men	29
	Women	9
Type of diabetes (n)	Type 1	18
	Type 2	20
History of insulin use (years), range		4–32
Diagnostic examination (n)	Pathological examination only	23
	Pathological examination+CT	4
	Pathological examination+MRI	3
	Pathological examination+CT, blood test	2
	Pathological examination+CT, ultrasonography	1
	Pathological examination+CT, PET	1
	Pathological examination+CT, MRI, ultrasonography	1
	Pathological examination+CT, blood test, ultrasonography	1
	Pathological examination+ultrasonography	1
	Pathological examination+blood test	1
Treatment (n)	Surgical excision	11
	Change of insulin injection site	9
	Surgical excision+change of insulin injection site	2
	Change insulin injection site+instructions on how to store insulin products	1
	Ointment application	1
	NR	14

Abbreviations: CT, computed tomography; MRI, magnetic resonance imaging; PET, positron emission tomography; NR, not reported.

**Table 3:** Morphological characteristics of insulin-derived amyloidosis

Location (n)	Abdomen	23
	Upper limbs	4
	Abdomen+upper, lower extremities	2
	Abdomen+upper extremities	1
	Abdomen+lower extremities	1
Size (mm), range		10–130
Shape (n)	Aril form	2
Color (n)	Reddish brown	1
	Brown to purplish-red	1
	Grayish-white	1

**Table 4:** Subjective symptoms, patient awareness, and reasons for medical consultation

Authors	Year	Subjective symptoms	Patient awareness	Reasons for medical consultation
Nagase T, et al.	2009	Patient had been aware of the nodules for several months	Because the region was easy to palpate and painless, it was regularly used for injection	Poor glycemic control
Takao M, et al.	2012	Patient had been aware of induration for approximately 2 years	NR	Brownish induration appeared gradually, so the patient consulted a local doctor
Kudo-Watanuki S, et al.	2013	Patient was aware of the presence of the nodules	Patient chose this location to avoid injecting near their appendectomy scar	Diabetic foot gangrene
Nagase T, et al.	2014	NR	NR	Diabetic retinopathy
Nagase T, et al.	2014	NR	NR	Poor glycemic control
Nagase T, et al.	2014	NR	NR	Hypoglycemia with lethargy
Nagase T, et al.	2014	NR	NR	Cerebral infarction
Nagase T, et al.	2014	NR	NR	Diabetic foot gangrene
Yabe S, et al.	2015	Patient was aware of the presence of the nodules	NR	Brownish induration appeared gradually, so the patient visited his local doctor
Mangla, A, et al.	2016	Patient noticed rhizoids; the lesions were painful, and gradually increased in size, ulcerated, and sometimes bled	NR	NR
Okamoto T, et al.	2017	Patient had been aware of a mass over their lower abdomen for 6 years	Easy to grasp for injection	Hospitalized for hypoglycemia
Higuchi T, et al.	2017	Patient noticed nodules over their right and left lower abdomen	Easy to grasp and painless when injecting	Poor glycemic control
Miyake M, et al.	2018	Two months before his first visit, the patient presented with subcutaneous rigidity in his lower left abdomen	NR	Patient was seen by a local dermatologist
Utsunomiya R, et al.	2018	Patient was aware of the presence of the nodules	Continued to inject in the same location because of the ease of pinching during injection	Patient suspected an insulin ball and went to see his doctor
Tomita S, et al.	2018	Patient became aware of a mass in their lower umbilicus 3 years previously, and this gradually increased in size; no pain	NR	A gynecologist was consulted because a mass was found
Tachibana M, et al.	2019	Patient was aware of the presence of the nodules	Continued to inject in the same location because of the ease of pinching during injection	Gradual increase in the size of the skin mass
Kitano Y, et al.	2019	Patient became aware of a skin mass over their left lower abdomen 3 years previously, and this gradually increased in size	NR	NR
Nagase T, et al.	2021	NR	NR	Hypoglycemia with lethargy
Chen MC, et al.	2020	Patient became aware of two advanced skin tumors, on both sides of their umbilicus, 5 years previously	NR	NR
Samlaska, et al.	2020	Patient had first noticed the mass 10 years previously	Patient was aware of the presence of a mass but thought there was no further enlargement and continued to inject	NR
Miyata T, et al.	2020	Patient became aware of a nodule at the site of a subcutaneous injection over the abdomen 10 years previously	NR	Patient was seen because of the gradual growth of the nodule

Abbreviations: NR, not reported.
